# Stem cells for neonatal stroke- the future is here

**DOI:** 10.3389/fncel.2014.00207

**Published:** 2014-07-25

**Authors:** Cindy T. J. van Velthoven, Fernando Gonzalez, Zinaida S. Vexler, Donna M. Ferriero

**Affiliations:** ^1^Laboratory of Neuroimmunology and Developmental Origins of Disease, University Medical Center UtrechtUtrecht, Netherlands; ^2^Departments of Pediatrics and Neurology, UCSFSan Francisco, CA, USA

**Keywords:** mesenchymal stem cells, neonatal stroke, BDNF, hypoxia-ischemia, brain, outcomes

## Stem cells

In recent years stem cell therapy has emerged as a potential treatment for neonatal ischemic brain injury. The efficacy of cell- based therapies in restoring damaged brain tissue has been tested in a multitude of models for different CNS diseases. Several different stem and progenitor cell populations have been utilized as cell-based therapy, including neural stem cells, embryonic stem cells, human umbilical cord blood cells (HUBCs), hematopoietic stem and progenitor cells, and mesenchymal stem cells (MSCs). Most stem cell types appear to enhance recovery to some extent (Pimentel-Coelho and Mendez-Otero, 2010). However, because of their low immunogenicity, availability and positive results obtained from preclinical studies, MSCs are a particularly promising candidate to repair the devastating effects that are associated with neonatal stroke. MSCs were first isolated and identified in bone marrow, but can now be isolated from many tissues, including adipose tissue, muscle, skin and extraembryonic tissues like the placenta, umbilical cord and Wharton's jelly. The latter sources are of particular interest for neonates that experience an ischemic event around the time of birth, at which time cells can be harvested and transplanted from an autologous source. MSCs derived from different sources have slightly different characteristics, but as of yet it is unknown whether this influences their therapeutic potential.

Our group and others have shown that administration of MSCs reduces lesion volume, provides positive effects on the white matter and improves motor function (van Velthoven et al., 2012). Numerous studies have been done under the premise that transplanted stem cells contribute to brain repair by directly replacing damaged or lost tissue. While there is evidence that transplanted cells undergo differentiation toward neuronal lineages, improved outcomes have been observed even when survival of transplanted cells is low and engrafted cells are absent. This suggests that rather than replacing damaged cells, transplanted cells may improve outcome via indirect mechanisms. For example, MSCs have been shown to secrete many factors that can influence important processes like apoptosis, neurogenesis, angiogenesis and synaptogenesis.

## Mechanisms of stem cell mediated brain repair—cellular aspects

Although results from experimental animal models using cell-based therapies to treat ischemic brain injury are very promising, there are several issues that need to be addressed. For example, the therapeutic time window for stem cell therapy is not well-defined. This has to do with the fact that several models for ischemic brain injury are being used and several different stem cell types are being tested. Beneficial effects of stem cells have been observed when they are transplanted anywhere from 3 h to 10 days after onset of injury. In a study where MSCs were administered at 17 days post-injury, no beneficial effects were observed. These data indicate that stem cells might serve both a neuroprotective and a neuroregenerative role. This dual role is further underlined by results showing that apoptosis is reduced after transplantation of stem cells, while on the other hand, endogenous neurogenesis is enhanced. In a recent study, multiple injections of MSCs were shown to be more beneficial than a single injection. Moreover, the timing of injection may influence different repair processes. A first injection with MSCs at 3 days after injury stimulated cell proliferation and differentiation, while a second injection at 10 days stimulated axonal remodeling. Combined these results show that it is imperative to investigate via which mechanism MSCs and other stem cells exert their beneficial effects, since this will help identify more precise targets to improve both neuroprotection and regeneration.

The distribution of cells within injured brain after intravenous injection or other modes of delivery, and potential adverse effects of exogenous administration, have not been thoroughly explored. The fact that MSCs administered via intranasal delivery migrate toward injured regions provides a promising practical avenue for such treatments.

The temporal-spatial effects of stem cells often depend on the animal model of brain injury and the source or manipulation of these cells. Stem cells often home to different regions of the brain, with emphasis on injured or inflamed areas. Once in the circulation, these cells can also differentiate into a wide variety of cell types, including immune cells, endothelial progenitors, and neuronal or glial cell types (Liu et al., 2014). In other organs, MSCs persist for as long as several months. While growth factors play a role in the efficacy of exogenous stem cells and endogenous precursor cells, it is likely that stem cells act as a trophic factor factory enhancing neuroprotective and endogenous neurogenic capacity. In this sense a therapeutic strategy in which application of stem cells is combined with growth factor administration to target a specific event may be more beneficial in combatting the events that cause progression of brain injury after stroke but, again, the timing of administration would ultimately determine the added repair efficacy in the brain. Mobilization of circulating endothelial progenitor cells (EPC) may be another key mechanism via which stem cells exert beneficial effects, as adequate blood supply is essential for effective repair of damaged tissue.

## Mechanisms of stem cell mediated brain repair—molecular aspects

The evidence for the role of growth factors on cell proliferation and tissue repair after stem cell transplantation includes increased expression of a number of factors that lead to proliferation and integration of endogenous cells into neural networks, while also enhancing angiogenesis. As mentioned, HUBCs have demonstrated an ability to express neuronal, astrocytic, and oligodendrocyte markers *in vitro*, but a very small percentage of engrafted HUCBs or MSCs survive and express neuronal phenotypes. Many grafted cells remain undifferentiated far from the lesion, where these undifferentiated stem/progenitor cells can directly release growth and trophic factors, or promote the release of such factors from host brain cells. For example, MSCs have been shown to produce trophic factors that induce survival and regeneration of host neurons, while also producing extracellular matrix molecules, resulting in functional improvement at both early and late time points after brain injury. Some of these factors include brain-derived neurotrophic factor (BDNF), glial -derived neurotrophic factor (GDNF), nerve growth factor (NGF), hepatocyte growth factor (HGF), vascular endothelial growth factors (VEGF), angiopoietin-1 (Ang-1) (Liu et al., 2014).

Thus, improvements in histological injury and neurological function in animals after stem cell transplantation are poorly understood but appear to be secondary to effects on the microenvironment in the injured brain. This could enhance host cell connections by improving synaptic plasticity and axonal connections, while also increasing neurogenesis and angiogenesis. For example, VEGF and its receptor are shown to rebound after middle cerebral artery occlusion (MCAO) in animals treated with MSCs. Similarly, cytokines and growth factors such as macrophage inflammatory protein 1 (MIP-1), matrix metalloproteinases (MMPs), erythropoietin receptor and tumor necrosis factor receptor are increased after MSC injection (Yang et al., 2010). This involves rapid change, occurring by 1 week after stroke, with continued elevation of levels at 4 weeks. Immune modulation may also play a significant role in this beneficial response. There is still much to be learned about how stem cell therapies may regulate innate and acquired immunity. Grafted cells often remain in the spleen and other organs and attenuate inflammatory mediators. This may result in increased bioavailability of soluble factors such as GDNF and BDNF.

Effects of stem cell therapy on angiogenesis and blood vessel remodeling may be particularly important following stroke. Transplantation of HUCB-derived MSCs increased the formation of new blood vessels and increased cortical blood flow in a rat model of MCAO. *In vitro* studies of MSCs have demonstrated production of a number of angiogenic factors, including VEGF, Ang-1, IGF-1, and G-CSF. This leads to enhancement of endothelial cell proliferation and recruitment of endogenous progenitor cells, promoting the growth of new vessels. Angiogenesis is mainly regulated by the VEGF/VEGF receptor and the angiopoietin/Tie-2 signaling pathway, and the expression of Tie-2 was increased after HUCB transplantation.

All of these data have led to the alternative strategy of combining cell-based therapy and gene delivery. In a hind limb ischemia model, the combination of intravenous infusion of EPCs over-expressing VEGF with local SDF-1 application was more efficient in improving local blood supply than either alone. Transplantation of EPCs over-expressing IGF-1 has led to inhibition of cardiac apoptosis, enhancement of cardiomyocyte proliferation, and increased capillary numbers in the peri-infarct area. Interestingly, VEGF over-expression in EPCs could increase the expression of CXCR4, leading to further enhancement of EPC homing. However, VEGF is also known to enhance vascular permeability in the brain early after an ischemic insult, and genetically modified MSCs expressing VEGF may actually increase functional deficits. On the other hand, hypoxia preconditioning enhances VEGFR2 expression on EPCs, and accordingly, augments the neovascularization efficacy of EPCs after administration. In addition, pre-incubating EPCs with SDF-1 enhances their pro-angiogenic potential in hind limb ischemia.

Studies using stem cells that over-express other neurotrophic factors, such as BDNF, have also shown promise for treating ischemic brain injury (Chen et al., 2013). BDNF is secreted by brain cells and induces neuroprotection, while promoting the synaptic and axonal plasticity associated with learning, memory, and sensorimotor recovery, and increasing newly born neurons in several regions of the brain. Administration of BDNF modified MSCs has produced therapeutic benefits in a rat model of transient MCAO. Transplantation of BDNF gene-modified human MSCs results in increased BDNF levels in the ischemic lesion and stronger therapeutic effects than MSCs alone. BDNF secreted by MSCs may protect against hyperexcitability and modulate neuronal excitability via downregulation of the potassium-chloride co-transporter KCC2. BDNF may also modulate vascular permeability and BBB breakdown, and early effects on the brain may be secondary to changes in cerebral edema. Benefit has also been demonstrated in a spinal cord injury model with improved functional outcome and enhanced sprouting of raphé–spinal axons. In addition, reduction in ischemic lesion volume and improved functional outcome after stroke in the rat has been seen following treatment with human MSCs genetically modified to express GDNF. FGF-2-modified MSCs also significantly reduced infarct volume 14 days after MCAO.

Overall, stem cell therapy for brain repair after stroke and other neurological conditions has shown benefit and may be closer than we think.

### Conflict of interest statement

The authors declare that the research was conducted in the absence of any commercial or financial relationships that could be construed as a potential conflict of interest.
